# A Case of Gastric Atypical Lipomatous Tumor/Well‐Differentiated Liposarcoma With Endoscopic Morphological Changes

**DOI:** 10.1002/deo2.70146

**Published:** 2025-05-22

**Authors:** Rika Omote, Shizuma Omote, Hiroshi Sonobe, Ryosuke Hamano, Tatsuya Toyokawa, Shinya Otsuka, Takehiro Tanaka, Hiroyuki Yanai, Masaru Inagaki, Hidetaka Yamamoto

**Affiliations:** ^1^ Department of Diagnostic Pathology NHO Fukuyama Medical Center Hiroshima Japan; ^2^ Department of Internal Medicine Fukuyama Minami Hospital Hiroshima Japan; ^3^ Department of Surgery NHO Fukuyama Medical Center Hiroshima Japan; ^4^ Department of Gastroenterology NHO Fukuyama Medical Center Hiroshima Japan; ^5^ Department of Pathology and Oncology Okayama University Graduate School of Medicine, Dentistry and Pharmaceutical Sciences Okayama Japan; ^6^ Department of Pathology Okayama University Hospital Okayama Japan

**Keywords:** atypical lipomatous tumor, CDK4, MDM2, stomach, well‐differentiated liposarcoma

## Abstract

Atypical lipomatous tumor/well‐differentiated liposarcoma is a locally aggressive mesenchymal neoplasm composed of adipocytes and stromal cells. Gastric cases are exceedingly rare, and their malignant potential remains unclear. We report a case of a woman in her 60s who was found to have multiple submucosal tumor‐like lesions of the stomach. Over time, the tumors increased in size, requiring a laparoscopic partial gastrectomy. Histological examination revealed a tumor composed of both fatty tissue and fibrous stroma with nuclear atypia. Immunohistochemistry showed positivity for CDK4 and MDM2, and fluorescence in situ hybridization confirmed *MDM2* amplification, leading to a diagnosis of atypical lipomatous tumor/well‐differentiated liposarcoma. This case presented an unusual gastric manifestation, with multiple submucosal tumor‐like lesions on endoscopy and exhibiting progressive morphological changes over several years.

## 1 Introduction

Atypical lipomatous tumor/well‐differentiated liposarcoma (ALT/WDLPS) is a mesenchymal neoplasm composed of adipocytes and stromal cells with at least partial nuclear atypia, often following a locally advanced course [1]. ALT and WDLPS are both morphologically and genetically identical, with ALT referring to tumors in the extremities and WDLPS to those in the retroperitoneum or trunk. The malignant potential of ALT/WDLPS has been redefined in recent years. In the 4th edition of the World Health Organization (WHO) classification, it was categorized as indeterminate [2]. However, the 5th edition classifies ALT as indeterminate and WDLPS as malignant due to its strong local invasiveness and varying site‐dependent recurrence rates [1]. Although ALT/WDLPS is generally considered non‐metastatic, gastric cases are extremely rare, and its malignant potential in this location remains unclear. In this study, we report a case of ALT/WDLPS presenting as multiple multiple submucosal tumor (SMT)‐like lesions on endoscopy, with morphological changes observed over several years.

## 2 Case Report

### 2.1 Patient

**FIGURE 1:**
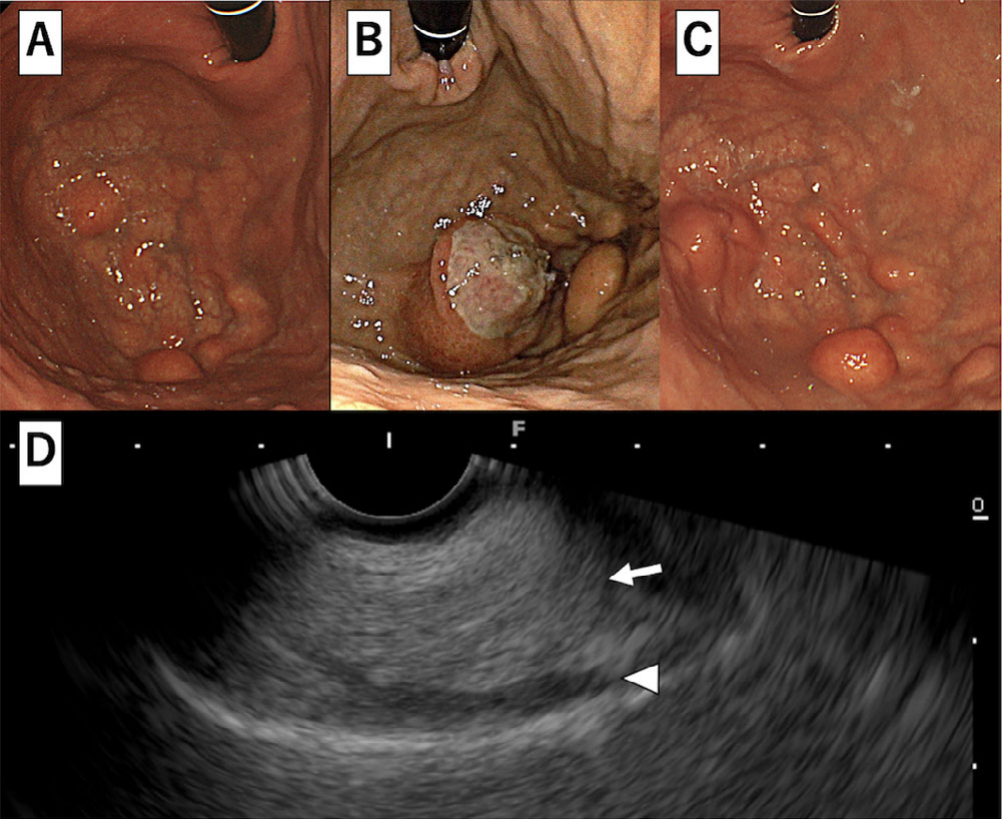
Esophagogastroduodenoscopy in X‐3 reveals multiple submucosal tumor‐like elevations (8–15 mm) in the greater curvature of the stomach (A). The submucosal tumor is significantly enlarged, with the largest reaching 3 cm in diameter and showing self‐destruction at the top (B). In year X + 2 months, the ulcer at the top disappeared (C). Endoscopic ultrasonography with a 7.5 MHz convex probe reveals a hyperechoic mass in the third layer of the stomach wall (D). The tumor is indicated by an arrow, and the muscularis propria layer is indicated by an arrowhead.

**FIGURE 2:**
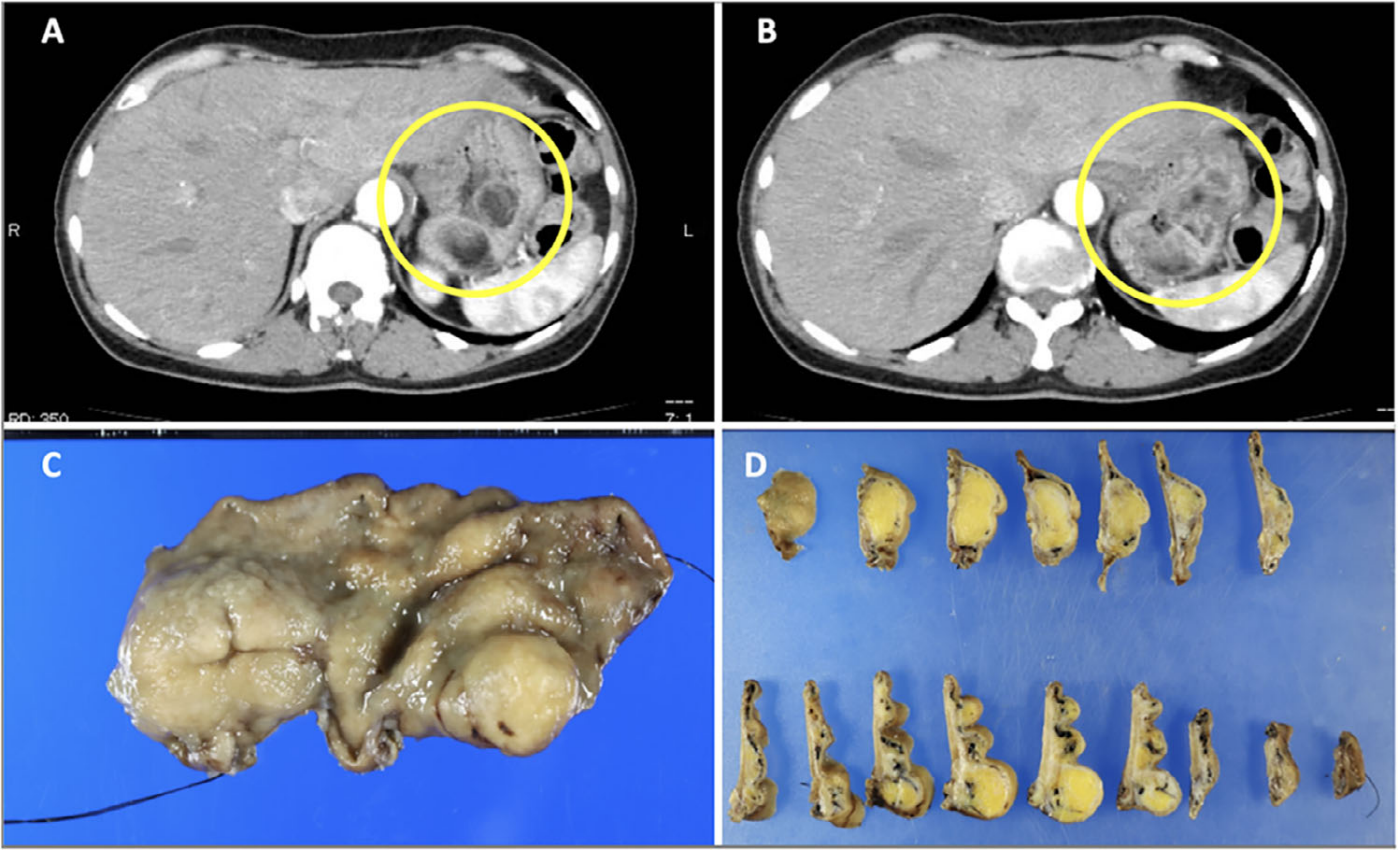
A low absorption area, thought to be fat, is observed in the submucosa of the fundus of the stomach on a contrast‐enhanced computed tomography scan (A, B). A faint soft‐tissue shadow, continuous with the same area, is also visible. The excised specimen shows a yellowish fatty tissue‐like nodule under the mucosa (C, D), corresponding to the nodule that appears nodular on the endoscopic cross‐section.

**FIGURE 3:**
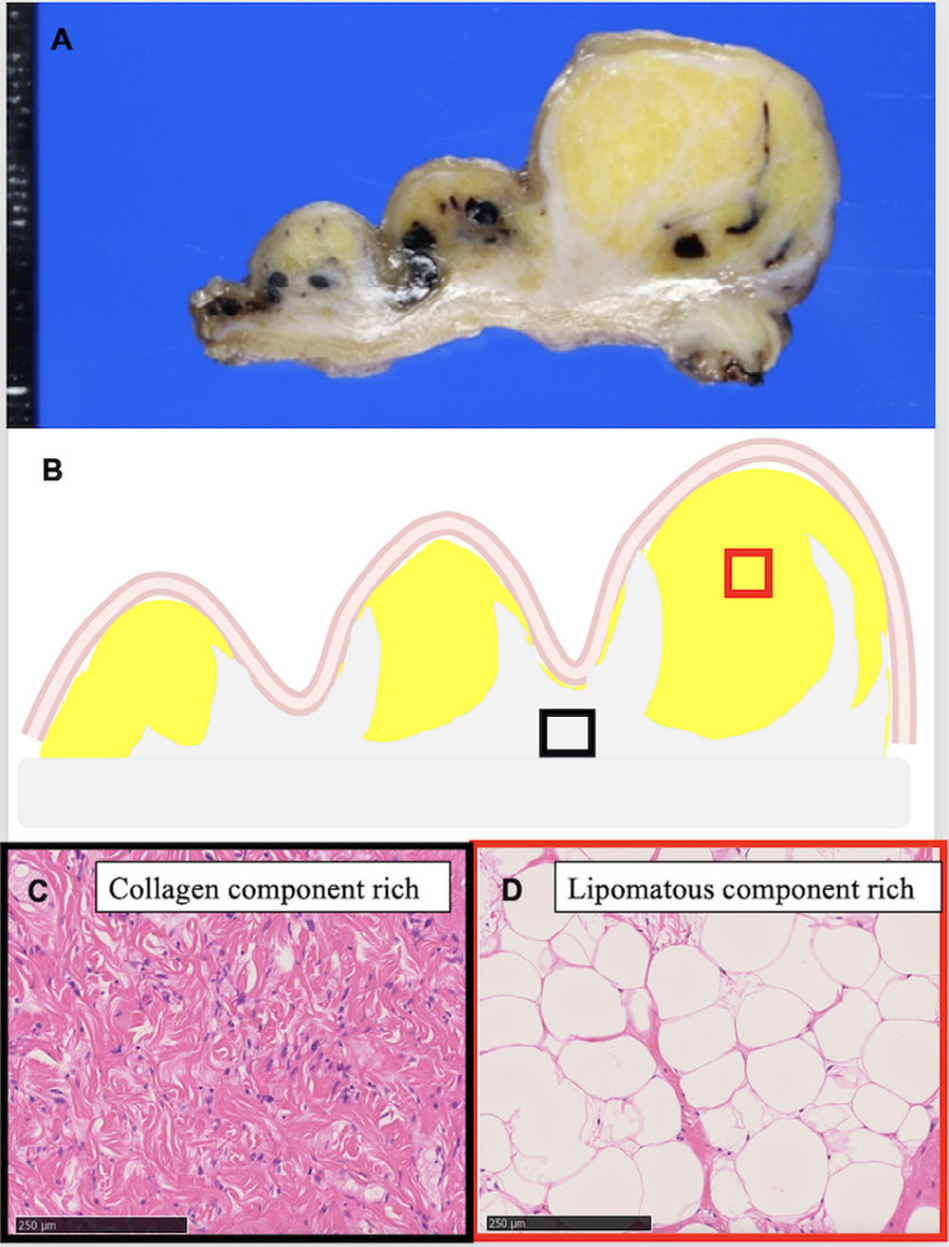
A grayish‐white tissue spreads under the mucosa in the form of intervening nodules. Histologically, the yellowish part of the nodular protuberances is rich in lipomatous components under the mucosa, while the grayish‐white area between the nodules contains abundant collagen components.

**FIGURE 4:**
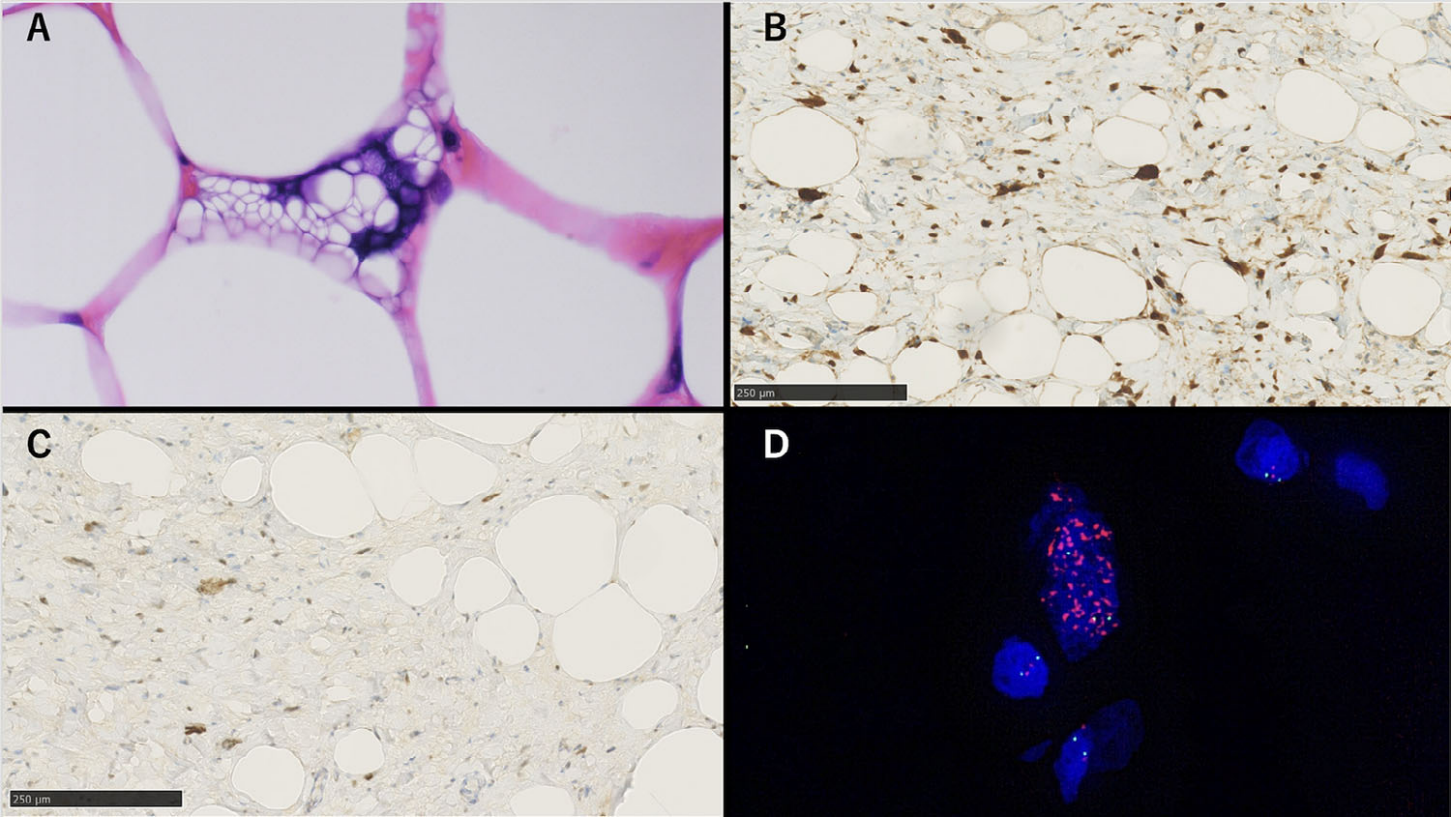
Some cells are identified as lipoblasts (A). Immunohistochemistry shows that the atypical cells are CDK4‐positive (B) and weakly to strongly positive for MDM2 (C). Fluorescence in situ hybridization analysis reveals *MDM2* gene amplification. The color coding of fluorescence in situ hybridization: *MDM2* (Texas Red; red signal), CEN12 (FITC; green signal), DAPI (nuclear staining) (D).

A woman in her 60s underwent an esophagogastroduodenoscopy (EGD) examination in year X‐3, revealing multiple SMT‐like elevations measuring 8–15 mm in the greater curvature of the stomach (Figure 1A). She was advised to return for a follow‐up examination after 6 months, but she did not comply. After 3 years, in year X, she consulted her family doctor due to dizziness. Blood tests revealed anemia with a hemoglobin level of 7.1 g/dL, leading to her referral to our hospital. A repeat EGD showed that the submucosal tumors previously detected in the fundus had increased significantly in size, with the largest tumor measuring 3 cm and a collapsed apex (Figure 1B). Additionally, a blood clot was attached to the top, suggesting bleeding from this site. At this time, a biopsy was performed on the lesion, yielding only necrotic tissue, making diagnosis impossible. Contrast‐enhanced computed tomography identified a tumor in the gastric fundus measuring approximately 6 cm in diameter, with a mixture of fatty and soft tissue components (Figure 2A,B). There was no significant lymph node enlargement on the computed tomography, nor were there findings that suggested metastasis to other organs. Subsequently, endoscopic ultrasonography revealed a high‐to‐low echoic mass predominantly in the submucosal tissue (Figure 1D). The urease test was negative. During a follow‐up EGD 2 months later, the ulcer at the top had resolved (Figure 1C). A fine‐needle aspiration biopsy by endoscopic ultrasonography was performed but yielded insufficient results for a definitive diagnosis. A laparoscopic partial gastrectomy was performed, given the tumor's progressive growth and the unresolved concern for malignancy. Macroscopic examination of the excised specimen revealed a yellowish, fatty tissue‐like nodule beneath the mucosa, corresponding to the multiple SMT‐like elevations observed endoscopically and grayish‐white tissue extending between these nodules (Figure 2C,D). Histological analysis showed a predominant proliferation of adipocytes in the yellow nodules, while the gray‐white areas between the nodules revealed a marked proliferation of fibrous stroma and stromal cells (Figure 3). Additionally, several adipocytes and stromal cells exhibiting enlarged nuclei and prominent nuclear atypia, with lipoblast‐like cells, were also observed (Figure 4A). Immunohistochemistry revealed that these cells were CD34‐ and CDK4‐positive (Figure 4B), with varying positivity for MDM2 (Figure 4C), and negative for S‐100 and α‐SMA. Only background mast cells were c‐kit positive. Fluorescence in situ hybridization revealed *MDM2* amplification (Figure 4D). Based on these findings, this tumor was diagnosed as ALT/WDLPS. The histological assessment indicated that the tumor extended sequentially from the elevated lesion to the flat lesion, with atypical cells present at the resection margin, preventing the confirmation of curative resection. Following an in‐depth discussion with the patient and family, close post‐operative surveillance was agreed upon. After 2.5 years of postoperative follow‐up, no recurrence has been observed.

## 3 Discussion

Liposarcoma, a malignant tumor of mesenchymal origin, is one of the most common soft tissue sarcomas. However, gastric liposarcoma is extremely rare, and since it was first reported in 1941 [3], there have been 59 documented cases. Histologically, there were 23 cases of well‐differentiated, 12 cases of myxoid, eight cases of dedifferentiated, five cases of pleomorphic, four cases of mixed, and the other cases of unknown types. According to our search on PubMed for previous reports of ALT/WDLPS, there were 11 cases with endoscopic findings. Six cases showed a solitary SMT‐like morphology, and one case showed a polyp‐like morphology. Four cases of them showed ulceration at the tip of the lesion. There are no reports of cases with multiple SMT‐like lesions as in the present case (Table S1).

According to Kang et al., 30.8% (8/26) of gastric liposarcomas occur in the fundus, 23.1% (6/26) in the lesser curvature of the cardia, 15.4% (4/26) in the lesser curvature of the body, 11.5% (3/26) in the greater curvatures of the cardia and body, and 3.8% (1/26) in the gastroesophageal junction [4]. In this case, EGD revealed a unique morphology with multiple SMT‐like lesions predominantly in the greater curvature of the fundus and fornix. Owing to ulcer formation (Figure 1B), the patient was prescribed vonoprazan 20 mg/day. As a result, 2 months later (Figure 1C), the ulcer had improved. Endoscopically, when submucosal tumors are typically observed, the differential diagnosis includes gastrointestinal stromal tumors, leiomyomas, lipomas, and ectopic pancreas. However, it is highly uncommon for these lesions to present as multiple SMT‐like lesions, as in this case. Even in liposarcoma cases, no previous reports have documented multiple lesions to the extent of morphological representation observed here. On endoscopic ultrasonography, the area rich in fat components was emphasized, making it look like a solitary tumor. Computed tomography revealed that despite the endoscopic appearance of multiple SMT‐like lesions, each elevation was connected under the mucosa, forming a single mass. Immature adipose‐derived stem cells are thought to be the origin of liposarcoma and to differentiate in two directions: towards fat‐rich cells and fat‐poor cells. These two differentiation trends are considered the cause of the multiple SMT‐like morphologies. In this case, both a yellow, fat‐rich area and a white, fat‐poor area were observed, and the increased fat cells had formed a submucosal mass and might have caused the SMT‐like elevation. Liposarcoma is thought to be characterized by its high proliferative potential and its resulting pleomorphic features. When multiple SMT‐like lesions are detected endoscopically, additional imaging studies are required to confirm the extent and continuity of the lesion. Magnetic resonance imaging has a high ability to visualize fatty components. If magnetic resonance imaging had been performed preoperatively, it would have detected a heterogeneous distribution of fatty components, which would have been a major differentiator from lipomas that show only a uniform fat layer.

Morphologically, ALT/WDLPS can be broadly categorized into three subtypes: lipocytic (lipomatous), sclerotic, and inflammatory [6]. This case represents the lipocytic (lipomatous) subtype. The lipocytic (lipomatous) subtype is likely common in gastric ALT/WDLPS, but there are no reports that mention the ratio of the three subtypes. Amplification of *MDM2* and *CDK4* is consistently observed across the subtypes [7]. In this case, immunohistochemistry revealed CDK4 positivity and varying degrees of MDM2 positivity, ranging from weak to strong. Since MDM2 immunohistochemistry can also be positive in some benign fatty tumors [8], fluorescence in situ hybridization was performed to confirm *MDM2* gene amplification [7, 9], thereby verifying the diagnosis of ALT/WDLPS. Dedifferentiation in ALT/WDLPS occurs when a high‐grade component emerges synchronously or metachronously within a well‐differentiated tumor with distinct boundaries [10]. Compared to the WHO 4th edition [2], the WHO 5th edition expands the concept of dedifferentiation [1], defining it histologically as a transition from a highly differentiated liposarcoma to spindle‐shaped cells and pleomorphic lipomatous tumor. In this case, a fatty tissue‐differentiated component in the nodular part and the fibrous stroma with stromal cells mixed with fatty tissue in the nodular interstitial region were observed. MDM2 and CDK4 expression was observed in both areas, suggesting a continuum of lesion development. However, in the absence of malignant transformation and the undefined dedifferentiation phenomenon, the patient was diagnosed with a well differentiated liposarcoma.

Atypical cells were observed near the resection margin, preventing confirmation of a negative margin. Following extensive discussion with the patient and her family, a decision was made to proceed with rigorous postoperative observation. Given the rarity of gastric liposarcoma, no established protocol exists for additional postoperative treatment. Further documentation of case reports is essential to inform evidence‐based management strategies for these tumors.

## Ethics Statement

N/A

## Consent

The patient has given informed consent for this case report.

## Conflicts of Interest

The authors declare no conflicts of interest.

## Clinical Trial Registration

N/A

## Supporting information




**TABLE S1** Report of 11 cases of gastric liposarcoma with endoscopic findings.
