# Supplementary material for: A Case of Gastric Atypical Lipomatous Tumor/Well‐Differentiated Liposarcoma With Endoscopic Morphological Changes
Source: DEN Open. 2025 May 22;6(1):e70146. doi: 10.1002/deo2.70146 (PMC12098964; doi:10.1002/deo2.70146)
Supplement: Supplementary file 1 — TABLE S1 Report of 11 cases of gastric liposarcoma with endoscopic findings. [file DEO2-6-e70146-s001.docx]

# Supplementary Table 1.

Reported cases of gastric liposarcoma with available endoscopic findings

| Author (Year) | Age | Sex | Histologic Type | Ulceration | Endoscopic Appearance |
| --- | --- | --- | --- | --- | --- |
| Tepetes K (2007) | 68 | M | Well | No | Ex |
| Matone J (2016) | 76 | M | Well | Yes | SMT |
| Girardot-Miglierina A (2018) | 72 | M | Mixed | Yes | SMT |
| Kang WZ (2018) | 45 | F | Well | No | SMT |
| Kang WZ (2018) | 69 | M | Well | No | SMT |
| Reishi T (2019) | 73 | F | Dedifferentiated | No | Ex |
| Hector W Cure (2020) | 70 | F | Well | Yes | SMT |
| Cho JH (2022) | 67 | F | Dedifferentiated | No | SMT |
| Fei Liu (2023) | 66 | M | Dedifferentiated | No | Flat lesion |
| Baker WG (2023) | 71 | M | Dedifferentiated | Yes | Ulcerative mass |
| Kubo K (2025) | 72 | M | Myxoid | Yes | Polypoid lesion |

Abbreviations: Dedifferentiated, dedifferentiated liposarcoma; Well, well-differentiated liposarcoma; Mixed, mixed-type liposarcoma; Myxoid, myxoid liposarcoma.

SMT, submucosal tumor-like lesion; Ex, extrinsic compression-like lesion.
